# Proteomics Data Analysis for the Identification of Proteins and Derived Proteotypic Peptides of Potential Use as Putative Drought Tolerance Markers for *Quercus ilex*

**DOI:** 10.3390/ijms22063191

**Published:** 2021-03-21

**Authors:** Bonoso San-Eufrasio, Ezequiel Darío Bigatton, Victor M. Guerrero-Sánchez, Palak Chaturvedi, Jesús V. Jorrín-Novo, María-Dolores Rey, María Ángeles Castillejo

**Affiliations:** 1 Agroforestry and Plant Biochemistry, Proteomics and Systems Biology, Department of Biochemistry and Molecular Biology, University of Cordoba, UCO-CeiA3, 14014 Cordoba, Spain; z82samab@uco.es (B.S.-E.); ezequielbigatton@gmail.com (E.D.B.); b12gusav@uco.es (V.M.G.-S.); bf1jonoj@uco.es (J.V.J.-N.); b52resam@uco.es (M.-D.R.); 2 Agricultural Microbiology, Faculty of Agricultural Science, National University of Cordoba, CONICET, 5001 Cordoba, Argentina; 3 Molecular Systems Biology Lab (MOSYS), Department of Functional and Evolutionary Ecology, University of Vienna, Althanstrasse 14, A-1090 Vienna, Austria; palak.chaturvedi@univie.ac.at

**Keywords:** peptide markers, *Quercus ilex*, drought tolerance, targeted post-acquisition proteomics

## Abstract

Drought is one of the main causes of mortality in holm oak (*Quercus ilex*) seedlings used in reforestation programs. Although this species shows high adaptability to the extreme climate conditions prevailing in Southern Spain, its intrinsic genetic variability may play a role in the differential response of some populations and individuals. The aim of this work was to identify proteins and derived proteotypic peptides potentially useful as putative markers for drought tolerance in holm oak by using a targeted post-acquisition proteomics approach. For this purpose, we used a set of proteins identified by shotgun (LC-MSMS) analysis in a drought experiment on *Q. ilex* seedlings from four different provenances (viz. the Andalusian provinces Granada, Huelva, Cadiz and Seville). A double strategy involving the quantification of proteins and target peptides by shotgun analysis and post-acquisition data analysis based on proteotypic peptides was used. To this end, an initial list of proteotypic peptides from proteins highly represented under drought conditions was compiled that was used in combination with the raw files from the shotgun experiment to quantify the relative abundance of the fragment’s ion peaks with the software Skyline. The most abundant peptides under drought conditions in at least two populations were selected as putative markers of drought tolerance. A total of 30 proteins and 46 derived peptides belonging to the redox, stress-related, synthesis,-folding and degradation, and primary and secondary metabolism functional groups were thus identified. Two proteins (viz., subtilisin and chaperone GrpE protein) were found at increased levels in three populations, which make them especially interesting for validation drought tolerance markers in subsequent experiments.

## 1. Introduction

Holm oak (*Quercus ilex*) is the dominant tree species in natural forest ecosystems over the Western Mediterranean Basin, as well as in the agrosilvopastoral Spanish “*dehesa*”, which is environmentally, economically and socially important [1,2]. This species is highly adaptable to drought and to the high temperatures and irradiation typical of Southern Spain. However, the main cause of mortality in holm oak plantations is water deficiency, with drought stress acting as a major factor of decline [3,4]. This situation can be expected to worsen in a scenario of climate change where statistical models have predicted that 40% of the land areas with a high-density of *Q. ilex* will be unsuitable for its survival [5].

Studies on the genetic variability associated with both environmental factors and genotypes have shown high population variability and polymorphism in *Quercus* spp [6,7,8,9]. In addition, *Q. ilex* has been shown to exhibit high variability in traits associated with drought tolerance, both within and between populations [10]. Because this is a non-domesticated species with a very long-life cycle, it is not amenable to conventional plant breeding. Therefore, for management and conservation practices based on resilient, elite genotypes of holm oak trees to be effective, they must rely on a sound knowledge of their biology and molecular mechanisms of adaptation to adverse climatic conditions. The response of plants to stress-related situations may, in theory, be improved by characterizing their biodiversity and selecting elite genotypes based on specific molecular markers. This approach can be quite challenging with orphan species, such as holm oak, which has a still incompletely sequenced genome and largely unexplored molecular features [11,12,13,14,15,16,17,18,19,20,21]. Fortunately, omics approaches have enabled crucial advances in these directions. Thus, some multiomics studies have addressed *Q. ilex* [19]; also, a reference transcriptome for this species has been generated [16,17], and, more recently, the metabolome of the acorn was determined [22]. In any case, the greatest efforts have focused on the proteomics of *Q. ilex*. A number of proteomic studies have used 1D and 2D gel-based analysis to investigate drought tolerance in this species [11,12,23]. In addition, recent studies have addressed various aspects of its biology by using shotgun (LC–MS/MS) proteomic analysis [19,21,24]. In addition, species-specific improved databases, such as the recently compiled holm oak transcriptome database [16,17], and other sequenced *Quercus* species databases, such as those for *Q. robur* [25] and *Q. suber* [26], have substantially expanded available knowledge of holm oak biology.

Quantitative proteomics is providing increasingly powerful tools for identifying markers of complex traits. Thus, identifying target peptide signals against mass spectrometry libraries is an efficient method for protein identification and quantification [27]. Targeted proteomics, however, does not allow the identification of new proteins as it requires the prior measurement of the targeted proteins by discovery proteomics; rather, it is useful for detecting changes in the protein abundances from previously acquired information [28]. Therefore, this proteomics branch can be useful to characterize coordinated changes in protein abundance with a view to identifying or validating proteins as markers for specific traits. Recently, proteotypic peptides have proved useful for protein quantification [29].

In this work, we used a double strategy for proteins and peptides quantification by targeted post-acquisition data analysis against a species-specific *Q. ilex* database with a view to identifying proteotypic peptides of potential use as putative drought tolerance markers for holm oak. For this purpose, a set of raw data generated in a shotgun experiment performed after 17 and 24 days under drought conditions in *Q. ilex* seedlings from four different provinces in Andalusia was used. Based on previous studies of inter-population variability of this species [12,18,30,31], we selected two populations from the southeast (Cadiz, Granada) and two from the northwest (Huelva, Seville) of Andalusia, with the purpose of identifying changes in proteins and derived peptides persistent over time in response to drought in different populations. In this work, various proteins and peptides are proposed as putative markers of drought tolerance in holm oak that transcend not only the tolerant phenotype but also populations and are examined in biological terms.

## 2. Results

### 2.1. Qualitative and Quantitative Analysis of Drought Stress Responsive Proteins

**Figure 1:**
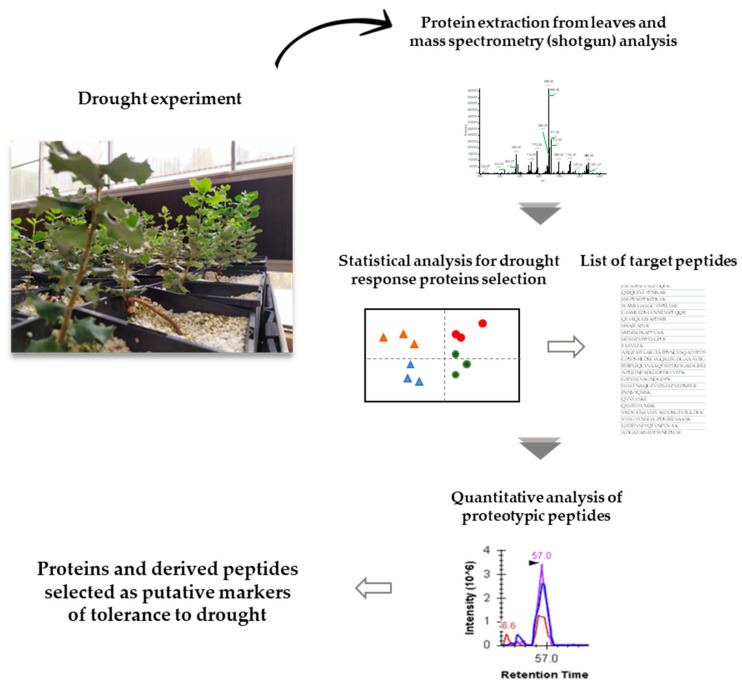
Schematic workflow for selection of putative markers of drought tolerance.

Shotgun analysis allowed a total of 4470 proteins to be identified in the *Q. ilex* leaf proteome (Supplementary Table S1; data are available via ProteomeXchange with identifier PXD023782) of which 2920 fulfilled the following criterion for confident identification: XCorr ≥ 2 and at least two different peptides per protein. An overall 2261 proteins were deemed variable in accordance with the following confidence criteria: (a) consistent presence in all replicates, (b) statistical significance with false discovery rate (FDR) < 0.05, and (c) drought/control ratio ≥ 2 and ≤0.5. In this group, 1692 proteins exhibited qualitative changes in at least one population and sampling time and 569 quantitative changes, 1683 being more abundant in droughted seedlings. The initial dataset was screened to select those variable proteins most markedly represented under drought conditions at both sampling times in each population. A total of 380 proteins were thus screened, of which 48 were present in at least two populations and deemed markers candidates. A schematic view of the workflow, as well as the details of the experimental design, are shown in Figure 1 and Supplementary Figure S1.

**Figure 2:**
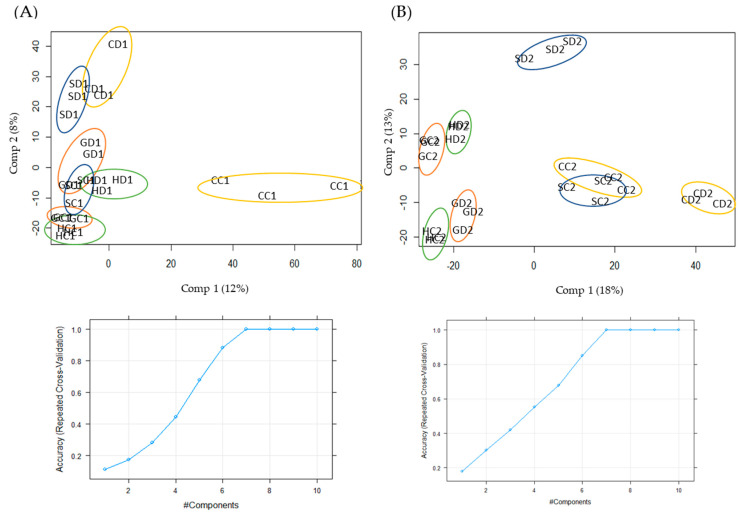
Partial least-squares discriminant analysis (PLS) of the entire dataset after 17 (**A**) and 24 days of drought (**B**) is shown in the upper part. The cumulative proportion of variance explained by components is shown below. C, Cadiz; G, Granada; H, Huelva; S, Seville. The letters following C, G, H, or S denote treatment (D, drought; C, control), the numbers before the underscore sampling time (1, 17 days; 2, 24 days) and that after it replicates (1, 2 or 3).

Multivariate analysis integrating the entire dataset is shown in Supplementary Figure S2A, where the first two components (25% of the total variability) separated populations. Hierarchical clustering was also performed and represented in a dendrogram (Supplementary Figure S2B), in which two main clusters were observed: one grouped Huelva and Granada populations, and the other grouped Seville and Cadiz, independently of the sampling time. Replicates of the same experimental condition (population, treatment and sampling time) were grouped, demonstrating reproducibility throughout the experiment. To check the effect of stress on the populations, the sampling times were analyzed separately by partial least-squares regression analysis (PLS-DA) (Figure 2). The first two components explained 20% (first sampling time) and 31% (second sampling time) of the total variability. Component 1 resolved Cadiz control plants, and component 2 resolved plants under drought for 17 days (first sampling) from control plants except in the Huelva population (Figure 2A). For the second sampling time (24 days), component 1 resolved the population pairs Huelva–Granada and Seville–Cadiz, and component 2 resolved treatments (Figure 2B). Based on this analysis, a clearer effect of drought treatment and populations can be appreciated at the second sampling time.

**Figure 3:**
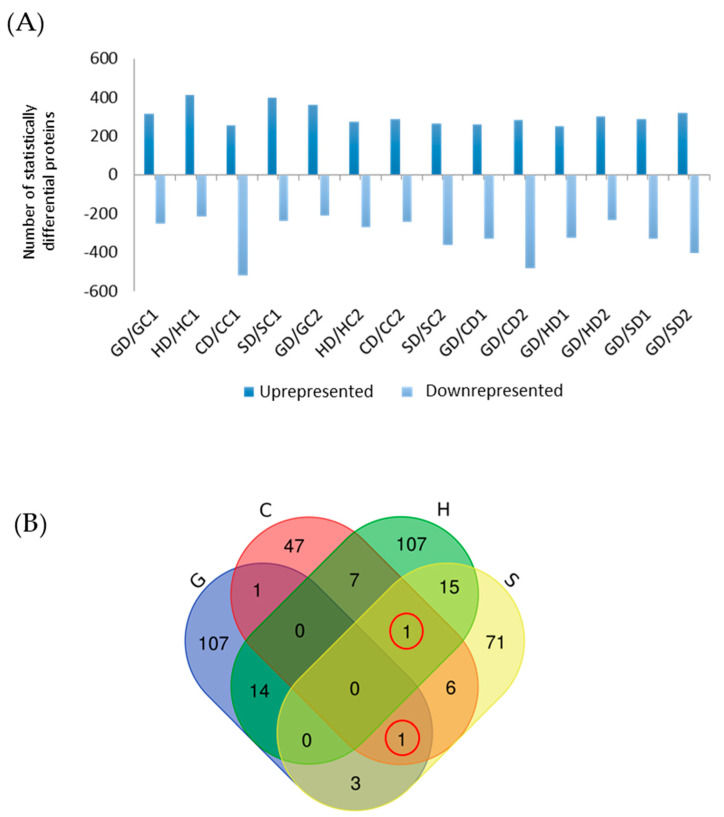
Variable proteins significantly (false discovery rate of 5%) uprepresented or downrepresented (twofold change) under drought conditions (**A**); C, Cadiz; G, Granada; H, Huelva; S, Seville. The letter following C, G, H or S denotes treatment (D, drought; C, control) and the number sampling time (1, 17 days; 2, 24 days). Venn diagram showing significantly up-represented proteins under drought conditions in each population (**B**).

Figure 3A shows the 2261 variable proteins significantly (FDR < 0.05) up- or down-accumulated (twofold change) in the drought group, and Figure 3B a Venn diagram of the 380 variable proteins among them. The Granada and Huelva populations exhibited the greatest number of unique variable proteins (107 each), followed by Seville (71) and Cadiz (47). Huelva and Seville shared the largest number of variable proteins (16), followed by Granada–Huelva (14), Huelva–Cadiz (8), Seville–Cadiz (8), Granada–Seville (4) and Granada–Cadiz (2). Only two proteins changed significantly by the effect of drought in three populations; thus, GrpE protein (qilexprot_13677) changed in Huelva, Seville and Cadiz, and subtilisin-like protease (qilexprot_25223) in Granada, Seville and Cadiz.

**Figure 4:**
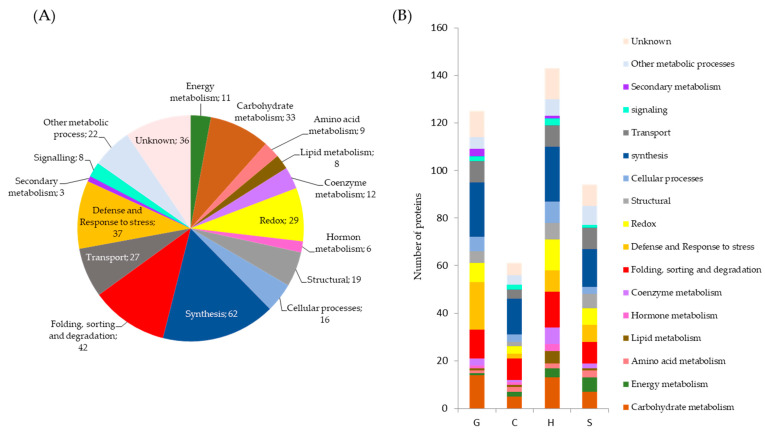
Functional categories of the 380 proteins. Total number of proteins significantly increasing in abundance after drought (**A**) and in each of the populations (**B**): C, Cadiz; G, Granada; H, Huelva; S, Seville.

The previous 380 variable proteins were characterized in functional terms by using Mercator [32] and GO enrichment (http://pantherdb.org/ accessed September 2020) for classification into 16 main groups (Figure 4A), namely: energy, carbohydrate, amino acid, lipid, hormone, coenzyme and secondary metabolism, other metabolic processes, cellular processes,-folding-sorting and degradation, synthesis (transcription/translation), structural, defense and response to stress, redox, signaling and transport. The best represented functional group was synthesis (62), followed by folding-sorting and degradation (42), defense and response to stress (37), carbohydrate metabolism (33) and redox (29), the previous five groups accounting for more than 50% of all identified proteins. Figure 4B compares the proteins whose abundance was altered by drought in each population as grouped by functional category.

### 2.2. Targeted Data Analysis for Selection of Peptides as Putative Markers of Drought Tolerance

**Figure 5:**
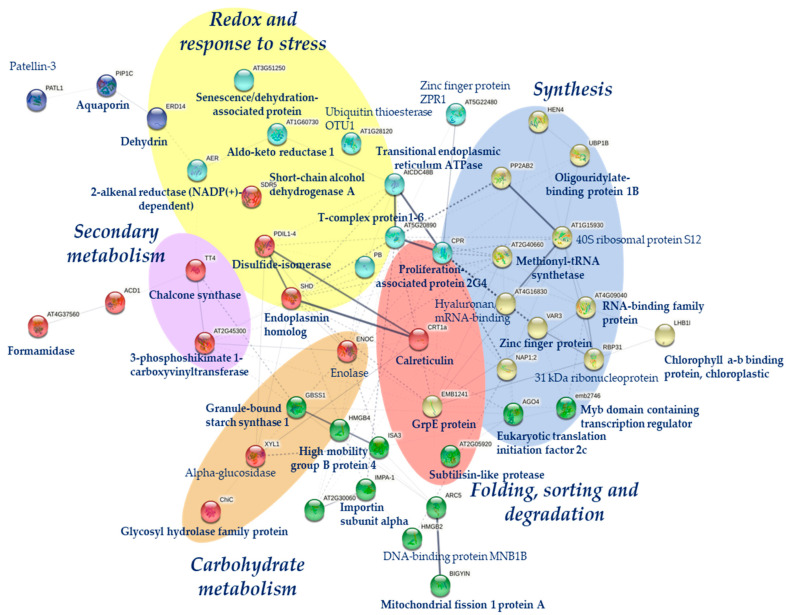
Analysis of the protein interaction network of the 48 proteins selected as putative markers of drought tolerance.

A list of proteotypic peptides derived from the 48 selected proteins was compiled for subsequent targeted analysis. The list, which included 219 proteotypic peptides with a charge state of +2 or higher, was used for quantification with the software Skyline as described in Section 4.5. As confirmed by Supplementary Figure S3, using the above-described library allowed 159 peptides to be successfully integrated with a robust, reproducible, high-quality peak shape in all three replicates (Supplementary Table S2). A statistical analysis of variance (ANOVA) on normalized data revealed 71 peptides comprising 32 different proteins were significantly better represented in droughted seedlings. Those peptides and proteins better represented in at least two populations (46 peptides from 30 proteins) were selected as putative markers of drought tolerance (Table 1). Supplementary Figure S4 illustrates protein and peptide quantification graphically. The most representative protein functions in the marker panel (Table 1) were synthesis and mRNA processing with 9 proteins. Seven proteins belonged to the redox and response to functional stress groups; 3 to the folding, sorting and degradation group; and 2 to the transport group. Other metabolic functional groups, such as carbohydrate metabolism (2), secondary metabolism (1), photosynthesis (1) and other processes (5), were also represented. The Huelva population was that exhibiting the greatest number of proteins, most of which belonged to the synthesis and mRNA processing group (7) and the stress-related and secondary metabolism group (6). Other proteins associated with energy and metabolism (viz., photosynthesis, carbohydrate metabolism and other cellular processes) were also represented (5). The second population as regards protein changes was Seville, with proteins of the metabolism (6), synthesis (4),-folding and degradation (3), and stress-related (3) groups. The Granada population exhibited smaller numbers of changing proteins, and only in the synthesis (5) and metabolism (3) groups. Finally, the Cadiz population was that exhibiting the least changes and mainly in stress-related (3), metabolic processes (2) and folding and degradation proteins (2). A protein–protein interaction network among the 48 proteins previously selected was performed using the web-tool STRING10 (http://string-db.org accessed January 2021) (Figure 5). A strong connection between proteins of synthesis and those of folding and degradation was observed.

## 3. Discussion

In this work, we used a double proteomic strategy for protein and peptide quantification in order to identify putative protein markers associated with drought tolerance in *Quercus ilex*. For this purpose, a dataset obtained by shotgun proteomics analysis of four *Q. ilex* populations from different provinces of Andalusia, Southern Spain, under severe drought stress [31] was analyzed by using a double strategy, combining shotgun protein quantification of proteins and target peptides with post-acquisition analysis of data based on proteotypic peptides. The populations were selected based on previous studies of *Q. ilex* variability between eastern populations and western ones [30,31], which demonstrated a relationship between tolerance and provenance. However, intrapopulation variability was also observed as corroborated by the study of morpho-physiological and biochemical parameters [31]. For this reason, the aim of this work was the search for putative drought tolerance markers that transcend not only the tolerant phenotype but also populations.

The typically high complexity of proteomes makes protein identification by mass spectrometry irreproducible as a result of precursor ions being selected stochastically. In addition, forest plants exhibit enormous biological variability that results in even poorer reproducibility among samples. Targeted proteomics analyses can be used to identify, characterize and quantify small sets of proteins previously selected by mass spectrometry analysis [33]. The few targeted proteomic studies conducted so far to identify markers of important traits have focused on crops, such as potato [34], apple [35], grapevine [36] and tomato [37,38]. Some have examined gluten profile [39,40], but even fewer have dealt with forest species. Although selected reaction monitoring (SRM) and its variants are the current gold standard for quantitative estimation of proteins, the recently data-independent acquisition (DIA) method is increasingly being used for targeted proteomics. Unlike existing alternatives, DIA extracts specific information from previously acquired data [41]. This method has been used in combination with proteotypic peptides to identify peptide markers of resistance to *Peyronellaea pinodes* in pea [42]. The use of proteotypic peptides for protein quantification recently proved more accurate than methods based on algorithms (usually on the intensity of the strongest ion peaks) [29].

As shown here, the shotgun technique, in combination with proteotypic peptides extracted from previously acquired data, has great analytical potential. While not a targeted proteomic strategy proper, this approach allows one to select peptides and proteins closely associated with specific traits. This requires processing a dataset compiled from a properly designed and conducted experiment (viz., one using a large enough number of replicates or individuals). In this work, we compiled a list of peptides and proteins potentially useful as putative markers of drought tolerance in *Q. ilex* that are briefly discussed in biological terms below. The proteins in the marker panel were differentially represented among populations, with the greatest numbers of changes found in the Huelva population, followed by Seville, Granada and Cadiz. Many of the selected proteins are involved in synthesis or degradation processes or, to a lesser extent, in metabolic processes, such as carbohydrate metabolism, photosynthesis and other metabolic reactions. Stress response, redox and secondary metabolism proteins were also well represented.

Changes in synthesis and degradation of proteins under stress conditions, such as drought, can be interpreted as a mechanism of adaptation through the installation of the translational apparatus and protein synthesis by recycling available amino acids in plants through protein degradation. Thus, plants respond to drought by synthetizing protective proteins and repairing or degrading damaged proteins [43]. Considering the general changes observed in the proteome in response to drought, synthesis (ribosomal and transcription) was the most represented group of proteins showing qualitative and quantitative changes, followed by folding and degradation category. Many of the proteins selected as putative markers here are involved in synthesis processes; such is the case, for instance, with translation initiation factor, zinc finger protein VAR3, RNA-binding protein, and methionyl-tRNA synthetase. The marker panel also included folding and degradation proteins, such as the chaperones calreticulin and GrpE protein, and serine protease subtilisin. 2DE-MSMS proteomic analysis previously revealed a similar response involving some of the previous proteins in *Q. ilex* [12,23] and *Q. robur* [44] under drought and suggested active metabolic adjustment to stress.

By contrast, degradation of starch in response to stress has been often associated with improved tolerance and potentially limited photosynthesis [45,46]. Sugars resulting from starch degradation, and other derivative metabolites, help plants grow under stress and function as osmoprotectants and compatible solutes to mitigate the adverse effects of stress [47], as found in droughted *Q. robur* [44]. Although environmental factors are known to have strong effects on the starch synthesis, their regulatory mechanisms remain unclear [48]. Some studies reported increased starch accumulation under stress, mainly in response to high salinity or cold [49,50,51]. In this work, two proteins of carbohydrate metabolism of the marker panel (viz., granule-bound starch synthase 1, which is chloroplastic/amyloplastic, and the glycosyl hydrolase family protein with chitinase insertion domain) were found at increased levels in, mainly, the Seville population. However, several starch degradation proteins not selected as putative markers were significantly increased in response to drought in some of the experimental conditions studied, including phosphoglucan water dikinase, α-glucan phosphorylase and α-amylase. Although apparently contradictory, this response may be related to the presence of different types of starch, whether permanent or transitory and that of isoforms involved in their synthesis and mobilization [48,52,53]. On the other hand, the photosynthetic machinery was seemingly unaffected; in fact, only a few photosynthesis proteins exhibited any changes, and only one (chlorophyll a-b binding protein) was included in the marker panel. This result is consistent with those of physiological studies on *Q. ilex* populations under severe drought in Seville, Granada and Cadiz, which exhibited no significant changes in photosynthetic pigments [31].

The broadest group of proteins and derived peptides selected as putative markers consisted of redox (2-alkenal reductase NADP-dependent, short-chain alcohol dehydrogenase A, disulfide-isomerase) and stress response proteins (endoplasmin, dehydrin, senescence/dehydration-associated protein and aldo-keto reductase). Some were closely associated with drought in several studies on the genus *Quercus* [4,44] or with biotic stress caused by *Phytophthora cinnamomi* [54]. Furthermore, a representative number of redox proteins not included in the marker panel have been identified as being increased to a greater or lesser extent in some of the conditions studied in response to drought, including glutathione S-transferase, glutathione peroxidase, thioredoxin, peroxidase, superoxide dismutase, lipoxygenase, among others. Our marker panel also included two enzymes involved in the shikimate–phenolic biosynthetic pathways, namely: chalcone synthase (CHS) and 3-phosphoshikimate 1-carboxyvinyltransferase. One of the potential roles of phenolic compounds is to scavenge harmful reactive oxygen species [55]. Consistent with our results, *Q. ilex* [31,56] and other *Quercus* spp. [57,58] were previously found to exhibit increased total levels of phenolics.

Finally, transport proteins, such as the water channel protein aquaporins, have been associated with plant tolerance of biotic and abiotic stresses, to which they respond by regulating the movement of water and small molecules through plasma membranes and vacuoles [59]. Based on a proteomics strategy involving the identification of proteotypic peptides, some transport proteins have been proposed as markers of tolerance to drought [60] and resistance to *Ascochyta* blight [42] in pea. The proteins were assumed to induce signaling and transport processes as mechanisms to maintain homeostatic equilibrium and cope with stress. The proposed putative markers included the importin subunit alpha, aquaporin and mitochondrial fission 1 protein A, although other transport and signaling proteins, such as 14-3-3-like protein, lipocalin, outer envelope pore protein (OEP), voltage-dependent anion-selective channel (VDAC) and translocase of chloroplast 90 protein, were also more represented under drought in some of the experimental conditions in this study.

Despite it is outside the scope of this work, based on our results, a clear distinction in response to drought among the populations studied cannot be postulated, for, which a greater number of populations covering a wider area must be included. However, attending to the high number of the proteins that showed changes in the Huelva population also observed in Seville, we could speculate on a similar response pattern to drought in both, perhaps due to geographic proximity. The fact that the proteomic profile of Cadiz is different from the rest may also have a geographical explanation as has already been described by Fernandez i Marti et al. [18], suggesting that the Guadalquivir Valley has played an important role in determining population divergence. To the authors’ knowledge, this is the first study aimed to identify proteins and derived peptides as putative markers of drought tolerance for a forest species, such as *Q. ilex*. Such markers may be useful with a view to selecting drought-tolerant genotypes or individuals.

## 4. Materials and Methods

### 4.1. Proteomic Dataset

The dataset used was compiled from the leaf proteome of *Q. ilex* seedlings in four different Andalusian populations, Southern Spain, namely: Granada (G), Huelva (H), Cadiz (C) and Seville (S). A detailed map of the Andalusian localizations from which samples were collected is shown in Supplementary Figure S5. All populations were under severe drought stress conditions, such as those imposed in summer under Mediterranean climate (Supplementary Table S3). Detailed information about specimen provenances (Supplementary Table S3), plant growth, stress conditions imposed, and physiological measurements can be found elsewhere [31]. Briefly, acorns were germinated and grown under greenhouse conditions in perlite, according to Simova-Stoilova et al. [23]. Severe drought was imposed by withholding water for 28 days at the 10-leaf stage under the following experimental conditions: 46/22 °C, 28 MJm^−2^/day, 41% HR [31]. Two different sampling times based on measured leaf physiological parameters were used for proteomic analysis. Thus, leaves were collected after 17 and 24 days of drought, corresponding to an average drop in chlorophyll fluorescence of 20% and 40%, respectively, in droughted seedlings relative to well-watered seedlings in all populations [31] (Supplementary Figure S6). These sampling times were selected as representative of an early and later stage of the response to drought with photosynthetically active leaves. Supplementary Figure S7 shows visual damage symptoms observed in the seedlings 25 days after a drought. Healthy leaves from different seedlings under different conditions as regards population, treatment and sampling time were collected, frozen in liquid nitrogen and stored at −80 °C for subsequent proteomic analysis.

### 4.2. Protein Extraction and Mass Spectrometry Analysis

Five fully expanded (photosynthetically active) leaves per plant from each population (G, H, C, S), treatment (control well-watered, control and drought) and sampling time (17 and 24 days) were crushed with liquid nitrogen and used for protein extraction. Proteins from three independent biological replicates (200 mg of fresh tissue each) were extracted with TCA/acetone-phenol [61], solubilized in a solution containing 7 M urea, 2 M thiourea, 4% (*w/v*) CHAPS (3 [(3-cholamidopropyl) dimethylammonio]-1-propanesulfonate), 0.5% (*w/v*) Triton X-100 and 100 mM DTT, and quantified by the Bradford method [62] using bovine serum albumin (BSA) as standard.

Shotgun analysis was performed by using 90 µg of BSA protein equivalents per sample that were prefractionated in SDS–PAGE according to Valledor and Weckwerth [63]. The resulting unique bands were excised from the gels and digested with proteomics-grade trypsin (Promega) to a final concentration of 12.5 ng/µL according to Romero-Rodríguez et al. [21]. Digested peptides were desalted by passage through C18 cartridges from Scharlau (Barcelona, Spain), and eluted peptides were vacuum dried and dissolved in a mixture of 70:30 (*v/v*) acetonitrile (ACN)/water containing 0.1% trifluoroacetic acid. Mass spectrometry analyses were conducted at the Proteomics Facility for Research Support Central Service (SCAI) of the University of Cordoba (Spain), using a Dionex Ultimate 3000 nano UPLC instrument from Thermo Scientific (San Jose, CA, USA) coupled to a nanoelectrospray ionization source and a trihybrid analyzer Thermo Orbitrap Fusion mass spectrometer, also from Thermo Scientific, operating in the positive ion mode. The specific settings used in the LC–MS/MS analyses are described elsewhere [42].

### 4.3. Protein Identification and Quantification

The raw data obtained from the MS analysis were processed with the software Proteome Discoverer v. 2.1.0.81 from Thermo Scientific. MS2 spectra were searched with the SEQUEST engine against the FASTA database obtained from the *Q. ilex* transcriptome [16,17]. Precursor mass tolerance was set at 10 ppm and fragment ion mass tolerance fixed at 0.1 Da. Only those ions with a charge state of +2 or greater were used. In silico peptide lists were generated by theoretical tryptic digestion, allowing up to two missed cleavages, carbamidomethylation of cysteines as a fixed modification and oxidation of methionine as a variable modification. Peptides were classified into protein groups according to the law of parsimony and filtered to FDR = 5% and XCorr ≥ 2.

Proteins were quantified in relative terms from the peak areas for precursor ions (specifically, the average of the three strongest peptide ion signals) from the identified peptides were used [64]. Protein values were then normalized by using a method that accounts for variability between runs to normalize relative protein abundance between samples, using the sum of the peak area values for each sample. Only those values consistently present in the three biological replicates were considered for further statistical analysis. Proteins were categorized by function by using the protein FASTA sequences in the software MERCATOR (https://www.plabipd.de/portal/mercator4 accessed August 2020) [30], an online tool for batch classification of proteins or gene sequences into Map-Man functional plant categories. In addition, nonannotated proteins were subjected to GO enrichment by using the Panther tool (http://pantherdb.org/ accessed September 2020).

The raw mass spectrometry data thus obtained were deposited on the ProteomeXchange Consortium (http://proteomecentral.proteomexchange.org on 25 January 2021) via the PRIDE partner repository [65] with the dataset identifier PXD023782.

### 4.4. Statistical Analysis of Data and Selection of Target Proteins

A partial least-squares-discriminant analysis (PLS-DA) of the entire dataset was performed to explain variance and correlation between the different variables, using the software RStudio v. 4.0.3 (Caret package v. 6.0-86 available at https://CRAN.R-project.org/package=caret) [66]. Target proteins were selected, and in silico data were analyzed by using the entire dataset provided by the shotgun analysis. All proteins selected met the following criteria: (a) the entire dataset was filtered by confidence parameters (score ≥ 2, at least 2 peptides per protein); and (b) they were the best represented in qualitative and/or quantitative terms (*p* ≤ 0.05) under drought conditions at both sampling times in at least two populations. Figure 1 illustrates the experimental workflow.

### 4.5. Targeted Post-Acquisition Data Analysis for Selection of Putative Peptides Markers

A list of target peptides generated from the proteotypic peptides (specific peptide sequences from the selected proteins as far as existing annotation information allowed for) was compiled. Proteotypic peptides were searched by aligning the sequences on the entire *Q. ilex* database, using the bioinformatics tools Bourne-again shell (Bash) (available at http://ftp.gnu.org/gnu/bash/ accessed November 2020) [67] and the BLAST protein (BLASTp) tool for the open source software operating system Ubuntu (available at https://www.exoscale.com/syslog/blast/ accessed November 2020). Proteotypic peptides were quantified by integrating the areas of the chromatographic fragment ion peaks in Skyline software (available at https://skyline.ms). The parameters used for relative quantification of MS1 were 0.055 m/z mass tolerance for the instrument, 0.5 m/z for library peak integration and a resolution of 120,000 at m/z 200. The integration peak and retention time (RT) for each peptide were checked by hand in order to confirm the reproducibility of ions among samples. Peptide values were subsequently assessed for statistical significance by using the external tool MS Stats in Skyline [68]. Data were normalized by equalizing intensity medians and then subjected to log2-transformation, after which ANOVA analysis was used to select the best-represented proteins among those exhibiting significant changes (*p* ≤ 0.05) under drought conditions. Supplementary Figure S4 provides a quantitative depiction of the proteins and precursor ions.

A protein interaction network of the selected proteins was generated by using the web tool STRING10 (http://string-db.org accessed January 2021). The protein homologs in *Arabidopsis* were analyzed by sequence BLASTing of the TAIR database (http://www.arabidopsis. org/Blast/index.jsp accessed January 2021), followed by application of STRING10 to develop a proteome-scale interaction network [69].

## 5. Conclusions

Methodologically, the proposed targeted strategy is aimed at identifying peptides associated with the response of *Q. ilex* to drought stress. As a supplement to shotgun analysis, using proteotypic peptides in addition to proteins allows putative markers enabling the identification of specific phenotypes, such as those best-resisting drought, to be selected. Our methodological workflow consisted of selecting those consistent and confident proteins whose proteotypic peptides were used for quantification. Of them, 46 peptides showed significant changes in response to drought stress in the same way that the protein they come from, which were proposed as putative markers.

Biologically, the results suggest that plants possess effective protective mechanisms for adaptation to drought through water loss prevention and protein protection and detoxification. However, small differences in response mechanisms may result in plant survival or adaptation to extreme conditions depending on the particular population or individual. As can be inferred from the composition of the marker panel, *Q. ilex* seedlings from four different populations responded differentially to drought, with the greatest number of changes being observed in the Huelva and Seville populations. Only two proteins (viz., the protease subtilisin and chaperone GrpE protein) were increased to a similar extent in three of the four populations. These proteins should be validated as biomarkers of drought tolerance in *Q. ilex* with further testing.

Our study constitutes a step forward in the molecular elucidation of this forest species. Advances in molecular techniques, including omics, in combination with physiological studies, can be expected to allow especially tolerant or resilient genotypes or individuals under stress conditions, such as those propitiated by a climate change scenario, to be selected in reforestation programs.

## Figures and Tables

**Table 1:** List of peptides and proteins selected as putative markers of tolerance to drought.

Protein ID	Peptide Sequences	Precursor *m/z*	Protein Description	Protein Function	Experimental Condition Showing Significant Change ^a^			
qilexprot_45247	DAWDTSVLVEMK	697,333	Granule-bound starch synthase 1, chloroplastic/amyloplastic	Carbohydrate metabolism			S	G
	FSFSDFSLLNLPDQFK	952,971					S	G
	QIEQLEVLYPNNAR	843,940					S	G
qilexprot_71384	SSEPESFPPKPDLVK	828,924	Glycosyl hydrolase family protein with chitinase insertion domain	Carbohydrate metabolism		H	S	
qilexprot_18873	WAMLGALGCVFPELLSR	968,491	Chlorophyll a-b binding protein, chloroplastic	Photosynthesis		H	S	
qilexprot_32784	GIAMLEDSLVNNTSSPLQQR	1087,046	Mitochondrial fission 1 protein A	Cellular processes	C		S	
	QLVEQCLEIAPDWR	878,934			C		S	
qilexprot_49492	SHAIEAFSR	509,256	T-complex protein 1 subunit beta	Cellular processes			S	G
qilexprot_42362	SMSESDKAPYVAK	714,834	High mobility group B protein 4	Cellular processes		H	S	
qilexprot_14200	SIDLSTVHYLSGPIR	552,964	Formamidase	Other metabolic process	C	H		
qilexprot_39395	FAEVLEK	418,228	3-phosphoshikimate 1-carboxyvinyltransferase	Other metabolic process		H		G
qilexprot_29542	AEGPATILAIGTATPSNCVSQADYPDYYFR	1083,173	Chalcone synthase	Secondary metabolism		H		G
	GPSDSHLDSLVGQALFGDGAAAVIIGADPDTK	1031,844				H		G
	IERPLFQLVSAAQTILPDSDGAIDGHLR	1011,205				H		G
qilexprot_49771	APLIDNPAFKDDPDLYVFPK	1138,082	Calreticulin	Folding, sorting and degradation		H	S	
qilexprot_25223	GIFVVCSAGNDGDFK	793,366	Subtilisin-like protease	Folding, sorting and degradation	C		S	G
qilexprot_13677	LSLLTNAQGEVVESLLPVLDNFER	1328,700	GrpE protein	Folding, sorting and degradation	C	H	S	
	INNSYQSISK	577,300			C	H	S	
qilexprot_55000	QVVLVSKE	451,268	2-alkenal reductase (NADP(+)-dependent)	Redox	C	H		
qilexprot_1533	QLSTDYCMAK	616,764	Short-chain alcohol dehydrogenase A	Redox	C		S	
	VRDVANAVLFLASDDSGFVTGLDLK	874,459			C		S	
qilexprot_55764	VVLGYLNSLVGPDSEELSAASK	1124,585	Protein disulfide-isomerase	Redox		H	S	
	LDDDVSFYQTVNPDVAK	963,456				H	S	
qilexprot_3345	ADGAFAISEDTWNEPLGR	974,952	Endoplasmin homolog	Response to stress		H	S	
	EVTEEEYTK	564,255				H	S	
	FYHSLAK	433,228				H	S	
	YLNFLMGLVDSDTLPLNVSR	1142,084				H	S	
	IAEEDPDEANDKDK	794,849				H	S	
qilexprot_72159	NKDEHETTTTTTPGGNEGAVESK	801,364	Dehydrin	Response to stress		H		G
	EEEMASEFEK	614,752				H		G
qilexprot_48029	ELAENLFPEDDTVSQTPTLQSSENVLVR	1044,179	Senescence/dehydration-associated protein AT3g51250	Response to stress	C		S	
qilexprot_8871	KGCTPSQLALAWVHHQGK	673,013	Probable aldo-keto reductase 1	Response to stress		H	S	
qilexprot_19464	VNWAYASGQR	576,300	Oligouridylate-binding protein	mRNA processing	C			G
	SVVELTNGSSEDGK	711,300			C			G
qilexprot_70616	LITVTASENPDSR	701,900	BnaC03g49780D protein	mRNA processing		H		G
qilexprot_26698	DKPESDGADLANK	680,319	Zinc finger protein VAR3, chloroplastic	mRNA processing		H	S	
	SVASNAIEWTGNASGSSVPDK	524,745				H	S	
qilexprot_2527	IEDIDAYAPK	567,784	Myb domain containing transcription regulator	Synthesis	C		S	
qilexprot_7552	IVDVCEIGDSFIR	761,800	Proliferation-associated protein 2G4	Synthesis		H	S	
	ALQLVVSECKPK	686,383				H	S	
qilexprot_56656	ISFSGIDGKPEDVLNPK	605,650	Probable methionyl-tRNA synthetase	Synthesis		H		G
qilexprot_70881	VQDTYDTELAGK	670,319	Eukaryotic translation initiation factor 2c	Synthesis		H		G
qilexprot_71168	EDENRLDEVGYDDVGGVR	679,305	Transitional endoplasmic reticulum ATPase	Synthesis		H		G
qilexprot_68980	EFFGSENNSLVSAQVIFHENPR	840,737	RNA-binding family protein	Synthesis		H	S	
qilexprot_68567	SPPINEVVQSGVVPR	789,400	Importin subunit alpha	Transport		H	S	
qilexprot_4392	GLYENSGGGANVVNHGYTK	968,957	Aquaporin	Transport		H		G

^a^*Q. ilex* population in which significant changes occurred under drought stress: C, Cadiz; G, Granada; H, Huelva; S, Seville.

## Data Availability

The raw mass spectrometry data were deposited to the ProteomeXchange Consortium (http://proteomecentral.proteomexchange.org on 25 January 2021) via the PRIDE partner repository with the dataset identifier PXD023782.

## Supplementary Materials

The following are available online at https://www.mdpi.com/1422-0067/22/6/3191/s1, Figure S1: Schematic workflow for selection of putative tolerance drought markers in *Q. ilex* seedlings by targeted post-acquisition proteomic analysis. C, Cadiz; G, Granada; H, Huelva; S, Seville, Figure S2: multivariate statistical analysis (PCA) of the entire dataset (A). Dendrogram shows hierarchical clustering of experimental conditions (B). C, Cadiz; G, Granada; H, Huelva; S, Seville. The letters following C, G, H, or S denotes treatment (D, drought; C, control), the numbers sampling time (1, 17 days; 2, 24 days), Figure S3: Representative MS1 fragment ion, retention times (RT) and normalized peak area of a target precursor ion given by the software Skyline, Figure S4: Quantitative protein (left) and abundance of precursor ions (right), Figure S5: Localization of all Andalusian Q. ilex provenances used in this study. Andalusia is delimited by the blue outline, Figure S6: Measurements of quantum yield of photosystem II (Fv/Fm) in dark-adapted leaves of Q. ilex of Andalusian provenances (Cadiz, C; Granada, G; Huelva, H; Seville, S) under drought conditions throughout the experiment. Values are means ± standard errors (SE). Arrows indicate the sampling times when the mean of leaf fluorescence had decreased by 20% after 17 days and 40% after 24 days relative to control seedlings, Figure S7: Visual damage symptoms in four Andalusian Q. ilex provenances (C, Cadiz; H, Huelva; S, Seville; G, Granada; the letters following C, H, S or G denotes treatment: D, drought; C, control) 25 days after drought treatment. Control seedlings did not show damage symptoms throughout the experiment. In contrast, some seedlings did show clear damage symptoms related to drought. Only asymptomatic seedlings were used in this study. Table S1: Raw data, normalized data and significant changes (FDR < 0.05) as calculated from the shotgun proteomics experiment, Table S2: List of proteotypic peptides derived from the 48 selected proteins, Table S3: Locations and environmental conditions of the four Q. ilex Andalusian populations.

## References

[1] Olea L., San Miguel-Ayanz A. The Spanish dehesa. A traditional Mediterranean silvopastoral system linking production and nature conservation. Proceedings of the 21st General Meeting of the European Grassland Federation.

[2] Abril N., Gion J.M., Kerner R., Muller-Starck G., Cerrillo R.M., Plomion C., Renaut J., Valledor L., Jorrin-Novo J.V. (2011). Proteomics research on forest trees, the most recalcitrant and orphan plant species. Phytochemistry.

[3] Crescente M.F., Gratani L., Larcher W. (2002). Shoot growth efficiency and production of *Quercus ilex* L. in different climates. Flora Morphol. Distrib. Funct. Ecol. Plants.

[4] Echevarria-Zomeno S., Ariza D., Jorge I., Lenz C., Del Campo A., Jorrin J.V., Navarro R.M. (2009). Changes in the protein profile of *Quercus ilex* leaves in response to drought stress and recovery. J. Plant. Physiol..

[5] Keenan T., Maria Serra J., Lloret F., Ninyerola M., Sabate S. (2011). Predicting the future of forests in the Mediterranean under climate change, with niche- and process-based models: CO2 matters!. Glob. Chang. Biol..

[6] Castro-Diez P., Villar-Salvador P., Pérez-Rontomé C., Maestro-Martínez M., Montserrat-Martí G. (1997). Leaf morphology and leaf chemical composition in three *Quercus* (Fagaceae) species along a rainfall gradient in NE Spain. Trees.

[7] Lumaret R., López de Heredia U., Soto A. (2009). Origin and genetic variability. Cork Oak Woodlands on the Edge: Ecology, Adaptive Management and Restoration.

[8] Valero-Galván J., Navarro Cerrillo R.M., Romero-Rodríguez M.C., Ariza-Mateos D., Jorrín-Novo J.V., Jorrín J.V., Vázquez J. (2010). Estudio de la respuesta al estrés hídrico en dos poblaciones de encina (*Quercus ilex* subsp. *ballota* (Desf.) Samp.) mediante una aproximación de proteómica comparativa basada en electroforesis bidimensional. Proteómica.

[9] Valero Galvan J., Valledor L., Navarro Cerrillo R.M., Gil Pelegrin E., Jorrin-Novo J.V. (2011). Studies of variability in Holm oak (*Quercus ilex* subsp. *ballota* [Desf.] Samp.) through acorn protein profile analysis. J. Proteom..

[10] Ramirez-Valiente J.A., Lorenzo Z., Soto A., Valladares F., Gil L., Aranda I. (2009). Elucidating the role of genetic drift and natural selection in cork oak differentiation regarding drought tolerance. Mol. Ecol..

[11] Jorge I., Navarro R.M., Lenz C., Ariza D., Jorrin J. (2006). Variation in the holm oak leaf proteome at different plant developmental stages, between provenances and in response to drought stress. Proteomics.

[12] Valero-Galvan J., Gonzalez-Fernandez R., Navarro-Cerrillo R.M., Gil-Pelegrin E., Jorrin-Novo J.V. (2013). Physiological and proteomic analyses of drought stress response in Holm oak provenances. J. Proteome Res..

[13] Jorrín-Novo J., Navarro-Cerrillo R.M. (2014). Variabilidad y respuesta a distintos estreses en poblaciones de encina (*Quercus ilex* L.) en Andalucía mediante una aproximación proteómica. Ecosistemas.

[14] Rico L., Ogaya R., Terradas J., Penuelas J. (2014). Community structures of N2 -fixing bacteria associated with the phyllosphere of a Holm oak forest and their response to drought. Plant. Biol..

[15] Rivas-Ubach A., Barbeta A., Sardans J., Guenther A., Ogaya R., Oravec M., Urban O., Peñuelas J. (2016). Topsoil depth substantially influences the responses to drought of the foliar metabolomes of Mediterranean forests. Perspect Plant. Ecol. Evol. Syst..

[16] Guerrero-Sanchez V.M., Maldonado-Alconada A.M., Amil-Ruiz F., Jorrin-Novo J.V. (2017). Holm Oak (*Quercus ilex*) Transcriptome. De novo Sequencing and Assembly Analysis. Front. Mol. Biosci..

[17] Guerrero-Sanchez V.M., Maldonado-Alconada A.M., Amil-Ruiz F., Verardi A., Jorrin-Novo J.V., Rey M.D. (2019). Ion Torrent and lllumina, two complementary RNA-seq platforms for constructing the holm oak (*Quercus ilex*) transcriptome. PLoS ONE.

[18] Fernández i Marti A., Romero-Rodríguez C., Navarro-Cerrillo R., Abril N., Jorrín-Novo J., Dodd R. (2018). Population Genetic Diversity of *Quercus ilex* subsp. *ballota* (Desf.) Samp. Reveals Divergence in Recent and Evolutionary Migration Rates in the Spanish Dehesas. Forests.

[19] Lopez-Hidalgo C., Guerrero-Sanchez V.M., Gomez-Galvez I., Sanchez-Lucas R., Castillejo-Sanchez M.A., Maldonado-Alconada A.M., Valledor L., Jorrin-Novo J.V. (2018). A Multi-Omics Analysis Pipeline for the Metabolic Pathway Reconstruction in the Orphan Species *Quercus ilex*. Front. Plant. Sci..

[20] Natali L., Vangelisti A., Guidi L., Remorini D., Cotrozzi L., Lorenzini G., Nali C., Pellegrini E., Trivellini A., Vernieri P. (2018). How *Quercus ilex* L. saplings face combined salt and ozone stress: A transcriptome analysis. BMC Genom..

[21] Romero-Rodriguez M.C., Jorrin-Novo J.V., Castillejo M.A. (2019). Toward characterizing germination and early growth in the non-orthodox forest tree species *Quercus ilex* through complementary gel and gel-free proteomic analysis of embryo and seedlings. J. Proteom..

[22] Lopez-Hidalgo C., Trigueros M., Menendez M., Jorrin-Novo J.V. (2021). Phytochemical composition and variability in *Quercus ilex* acorn morphotypes as determined by NIRS and MS-based approaches. Food Chem..

[23] Simova-Stoilova L.P., Romero-Rodriguez M.C., Sanchez-Lucas R., Navarro-Cerrillo R.M., Medina-Aunon J.A., Jorrin-Novo J.V. (2015). 2-DE proteomics analysis of drought treated seedlings of *Quercus ilex* supports a root active strategy for metabolic adaptation in response to water shortage. Front. Plant. Sci..

[24] Gomez-Galvez I., Sanchez-Lucas R., San-Eufrasio B., de Francisco L.E.R., Maldonado-Alconada A.M., Fuentes-Almagro C., Castillejo M.A. (2020). Optimizing Shotgun Proteomics Analysis for a Confident Protein Identification and Quantitation in Orphan Plant Species: The Case of Holm Oak (*Quercus ilex*). Methods Mol. Biol..

[25] Plomion C., Aury J.M., Amselem J., Alaeitabar T., Barbe V., Belser C., Berges H., Bodenes C., Boudet N., Boury C. (2016). Decoding the oak genome: Public release of sequence data, assembly, annotation and publication strategies. Mol. Ecol. Resour..

[26] Ramos A.M., Usie A., Barbosa P., Barros P.M., Capote T., Chaves I., Simoes F., Abreu I., Carrasquinho I., Faro C. (2018). The draft genome sequence of cork oak. Sci. Data.

[27] Gillet L.C., Navarro P., Tate S., Rost H., Selevsek N., Reiter L., Bonner R., Aebersold R. (2012). Targeted data extraction of the MS/MS spectra generated by data-independent acquisition: A new concept for consistent and accurate proteome analysis. Mol. Cell Proteom..

[28] Domon B., Aebersold R. (2010). Options and considerations when selecting a quantitative proteomics strategy. Nat. Biotechnol..

[29] Escandon M., Jorrin-Novo J.V., Castillejo M.A. (2021). Application and optimization of label-free shotgun approaches in the study of *Quercus ilex*. J. Proteom..

[30] Navarro-Cerrillo R.M., Ruiz Gomez F.J., Cabrera-Puerto R.J., Sánchez-Cuesta R., Palacios Rodriguez G., Quero Pérez J.L. (2018). Growth and physiological sapling responses of eleven *Quercus ilex* ecotypes under identical environmental conditions. Ecol. Manag..

[31] San-Eufrasio B., Sánchez-Lucas R., López-Hidalgo C., Guerrero-Sánchez V.M., Castillejo M.Á., Maldonado-Alconada A.M., Jorrín-Novo J.V., Rey M.-D. (2020). Responses and Differences in Tolerance to Water Shortage under Climatic Dryness Conditions in Seedlings from *Quercus* spp. and Andalusian *Q. ilex* Populations. Forests.

[32] Lohse M., Nagel A., Herter T., May P., Schroda M., Zrenner R., Tohge T., Fernie A.R., Stitt M., Usadel B. (2014). Mercator: A fast and simple web server for genome scale functional annotation of plant sequence data. Plant. Cell Environ..

[33] Rodiger A., Baginsky S. (2018). Tailored Use of Targeted Proteomics in Plant-Specific Applications. Front. Plant. Sci..

[34] Chawade A., Alexandersson E., Bengtsson T., Andreasson E., Levander F. (2016). Targeted Proteomics Approach for Precision Plant Breeding. J. Proteome Res..

[35] Buts K., Michielssens S., Hertog M.L., Hayakawa E., Cordewener J., America A.H., Nicolai B.M., Carpentier S.C. (2014). Improving the identification rate of data independent label-free quantitative proteomics experiments on non-model crops: A case study on apple fruit. J. Proteom..

[36] Riebel M., Fronk P., Distler U., Tenzer S., Decker H. (2017). Proteomic profiling of German Dornfelder grape berries using data-independent acquisition. Plant. Physiol. Biochem..

[37] Martin L.B., Sherwood R.W., Nicklay J.J., Yang Y., Muratore-Schroeder T.L., Anderson E.T., Thannhauser T.W., Rose J.K., Zhang S. (2016). Application of wide selected-ion monitoring data-independent acquisition to identify tomato fruit proteins regulated by the CUTIN DEFICIENT2 transcription factor. Proteomics.

[38] Mata C.I., Fabre B., Parsons H.T., Hertog M., Van Raemdonck G., Baggerman G., Van de Poel B., Lilley K.S., Nicolai B.M. (2018). Ethylene Receptors, CTRs and EIN2 Target Protein Identification and Quantification Through Parallel Reaction Monitoring During Tomato Fruit Ripening. Front. Plant. Sci..

[39] Bose U., Byrne K., Howitt C.A., Colgrave M.L. (2019). Targeted proteomics to monitor the extraction efficiency and levels of barley alpha-amylase trypsin inhibitors that are implicated in non-coeliac gluten sensitivity. J. Chromatogr. A.

[40] Bromilow S.N., Gethings L.A., Langridge J.I., Shewry P.R., Buckley M., Bromley M.J., Mills E.N. (2016). Comprehensive Proteomic Profiling of Wheat Gluten Using a Combination of Data-Independent and Data-Dependent Acquisition. Front. Plant. Sci..

[41] Meyer J.G., Schilling B. (2017). Clinical applications of quantitative proteomics using targeted and untargeted data-independent acquisition techniques. Expert. Rev. Proteom..

[42] Castillejo M.A., Fondevilla-Aparicio S., Fuentes-Almagro C., Rubiales D. (2020). Quantitative Analysis of Target Peptides Related to Resistance Against Ascochyta Blight (*Peyronellaea pinodes*) in Pea. J. Proteome Res..

[43] Vaseva I., Sabotič J., Šuštar-Vozlič J., Meglič V., Kidrič M., Demirevska K., Simova-Stoilova L., Neves D.F., Sanz J.D. (2012). The response of plants to drought stress: The role of dehydrins, chaperones, proteases and protease inhibitors in maintaining cellular protein function. Droughts: New Research.

[44] Sergeant K., Spiess N., Renaut J., Wilhelm E., Hausman J.F. (2011). One dry summer: A leaf proteome study on the response of oak to drought exposure. J. Proteom..

[45] González-Cruz J., Pastenes C. (2012). Water-stress-induced thermotolerance of photosynthesis in bean (*Phaseolus vulgaris* L.) plants: The possible involvement of lipid composition and xanthophyll cycle pigments. Environ. Exp. Bot..

[46] Cuellar-Ortiz S.M., De La Paz Arrieta-Montiel M., Acosta-Gallegos J., Covarrubias A.A. (2008). Relationship between carbohydrate partitioning and drought resistance in common bean. Plant. Cell Environ..

[47] Krasensky J., Jonak C. (2012). Drought, salt, and temperature stress-induced metabolic rearrangements and regulatory networks. J. Exp. Bot..

[48] Thalmann M., Santelia D. (2017). Starch as a determinant of plant fitness under abiotic stress. New Phytol..

[49] Kaplan F., Guy C.L. (2005). RNA interference of Arabidopsis beta-amylase8 prevents maltose accumulation upon cold shock and increases sensitivity of PSII photochemical efficiency to freezing stress. Plant. J..

[50] Yin Y.G., Kobayashi Y., Sanuki A., Kondo S., Fukuda N., Ezura H., Sugaya S., Matsukura C. (2010). Salinity induces carbohydrate accumulation and sugar-regulated starch biosynthetic genes in tomato (*Solanum lycopersicum* L. cv. ‘Micro-Tom’) fruits in an ABA- and osmotic stress-independent manner. J. Exp. Bot..

[51] Skirycz A., De Bodt S., Obata T., De Clercq I., Claeys H., De Rycke R., Andriankaja M., Van Aken O., Van Breusegem F., Fernie A.R. (2010). Developmental stage specificity and the role of mitochondrial metabolism in the response of Arabidopsis leaves to prolonged mild osmotic stress. Plant. Physiol..

[52] Wang S.J., Liu L.F., Chen C.K., Chen L.W. (2006). Regulations of granule-bound starch synthase I gene expression in rice leaves by temperature and drought stress. Biol. Plant..

[53] Prathap V., Tyagi A. (2020). Correlation between expression and activity of ADP glucose pyrophosphorylase and starch synthase and their role in starch accumulation during grain filling under drought stress in rice. Plant. Physiol. Biochem..

[54] Sghaier-Hammami B., Valero-Galvan J., Romero-Rodriguez M.C., Navarro-Cerrillo R.M., Abdelly C., Jorrin-Novo J. (2013). Physiological and proteomics analyses of Holm oak (*Quercus ilex* subsp. *ballota* [Desf.] Samp.) responses to *Phytophthora cinnamomi*. Plant. Physiol. Biochem..

[55] Sharma A., Shahzad B., Rehman A., Bhardwaj R., Landi M., Zheng B. (2019). Response of Phenylpropanoid Pathway and the Role of Polyphenols in Plants under Abiotic Stress. Molecules.

[56] Nogués I., Llusià J., Ogaya R., Munné-Bosch S., Sardans J., Peñuelas J., Loreto F. (2013). Physiological and antioxidant responses of *Quercus ilex* to drought in two different seasons. Plant. Biosyst. Int. J. Deal. All Asp. Plant Biol..

[57] Jafarnia S., Akbarinia M., Hosseinpour B., Modarres Sanavi S.A.M., Salami S.A. (2018). Effect of drought stress on some growth, morphological, physiological, and biochemical parameters of two different populations of *Quercus brantii*. Iforest Biogeosciences For..

[58] Ghanbary E., Tabari Kouchaksaraei M., Zarafshar M., Bader K.M., Mirabolfathy M., Ziaei M. (2020). Differential physiological and biochemical responses of *Quercus infectoria* and *Q. libani* to drought and charcoal disease. Physiol. Plant.

[59] Li J., Ban L., Wen H., Wang Z., Dzyubenko N., Chapurin V., Gao H., Wang X. (2015). An aquaporin protein is associated with drought stress tolerance. Biochem. Biophys. Res. Commun..

[60] Castillejo M.A., Iglesias-Garcia R., Wienkoop S., Rubiales D. (2016). Label-free quantitative proteomic analysis of tolerance to drought in *Pisum sativum*. Proteomics.

[61] Wang W., Vignani R., Scali M., Cresti M. (2006). A universal and rapid protocol for protein extraction from recalcitrant plant tissues for proteomic analysis. Electrophoresis.

[62] Bradford M.M. (1976). A rapid and sensitive method for the quantitation of microgram quantities of proteins utilizing the principle of protein-dye binding. Anal. Biochem..

[63] Valledor L., Weckwerth W., Jorrin-Novo J.K.S., Weckwerth W., Wienkoop S. (2014). An Improved Detergent-Compatible Gel-Fractionation LC-LTQ-Orbitrap-MS Workflow for Plant and Microbial Proteomics. Plant Proteomics. Methods in Molecular Biology (Methods and Protocols).

[64] Al Shweiki M.R., Monchgesang S., Majovsky P., Thieme D., Trutschel D., Hoehenwarter W. (2017). Assessment of Label-Free Quantification in Discovery Proteomics and Impact of Technological Factors and Natural Variability of Protein Abundance. J. Proteome Res..

[65] Perez-Riverol Y., Csordas A., Bai J., Bernal-Llinares M., Hewapathirana S., Kundu D.J., Inuganti A., Griss J., Mayer G., Eisenacher M. (2019). The PRIDE database and related tools and resources in 2019: Improving support for quantification data. Nucleic Acids Res..

[66] Kuhn M. (2020). Caret: Classification and Regression Training. https://CRAN.R-project.org/package=caret.

[67] Altschu S.F., Gish W., Miller W., Myers E.W., Lipman D.J. (1990). Basic Local Alignment Search Tool. J. Mol. Biol..

[68] Chang C.-Y., Picotti P., Hüttenhain R., Heinzelmann-Schwarz V., Jovanovic M., Aebersold R., Vitek O. (2012). Protein Significance Analysis in Selected Reaction Monitoring (SRM) Measurements. Mol. Cell Proteom..

[69] Suo J., Zhao Q., Zhang Z., Chen S., Cao J., Liu G., Wei X., Wang T., Yang C., Dai S. (2015). Cytological and Proteomic Analyses of *Osmunda cinnamomea* Germinating Spores Reveal Characteristics of Fern Spore Germination and Rhizoid Tip Growth. Mol. Cell Proteom..

