# Supplementary material for: Proteomics Data Analysis for the Identification of Proteins and Derived Proteotypic Peptides of Potential Use as Putative Drought Tolerance Markers for Quercus ilex
Source: Int J Mol Sci. 2021 Mar 21;22(6):3191. doi: 10.3390/ijms22063191 (PMC8003919; doi:10.3390/ijms22063191)

Supplementary Figure S1

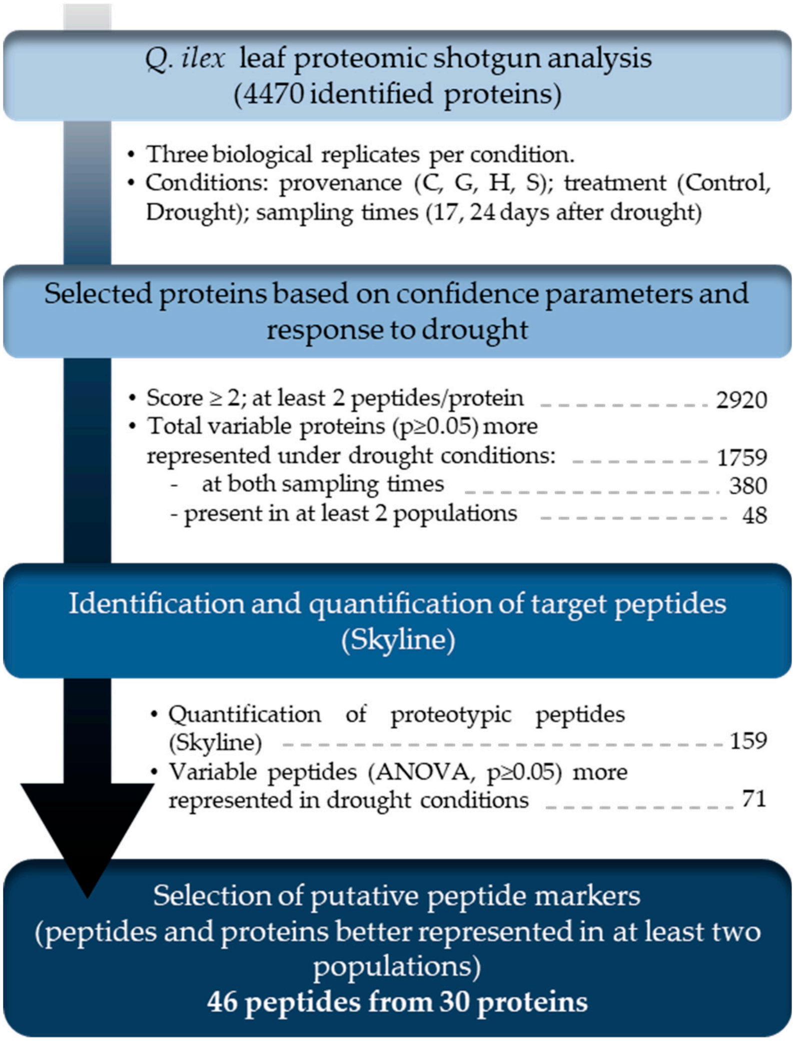

Supplementary Figure S2

(A)

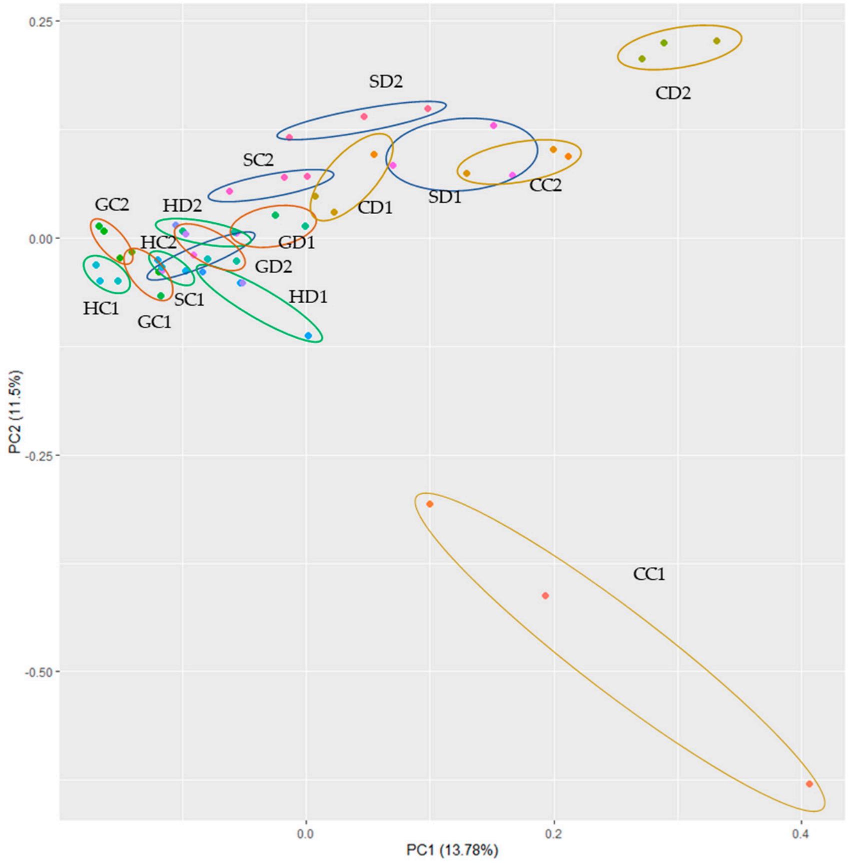

(B)

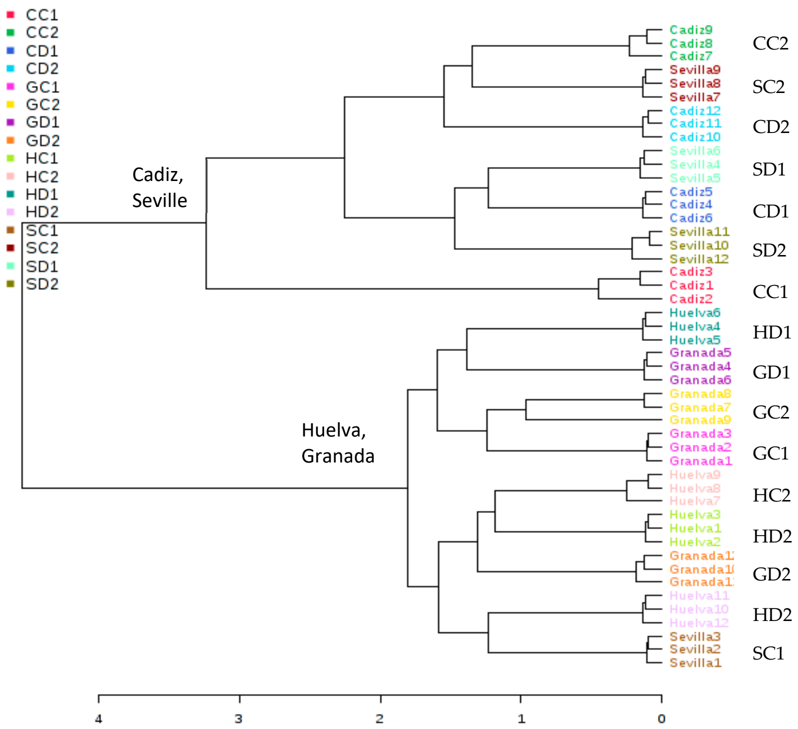

Supplementary Figure S3

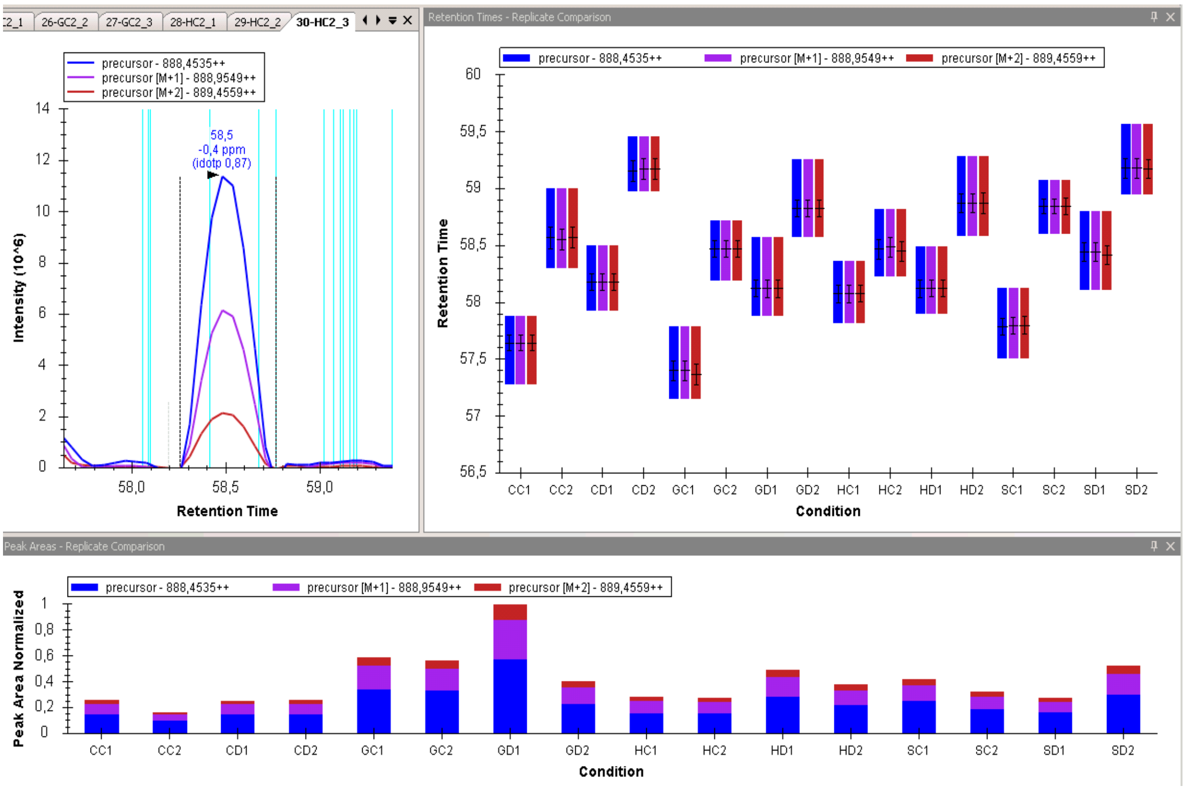

Supplementary Figure S4

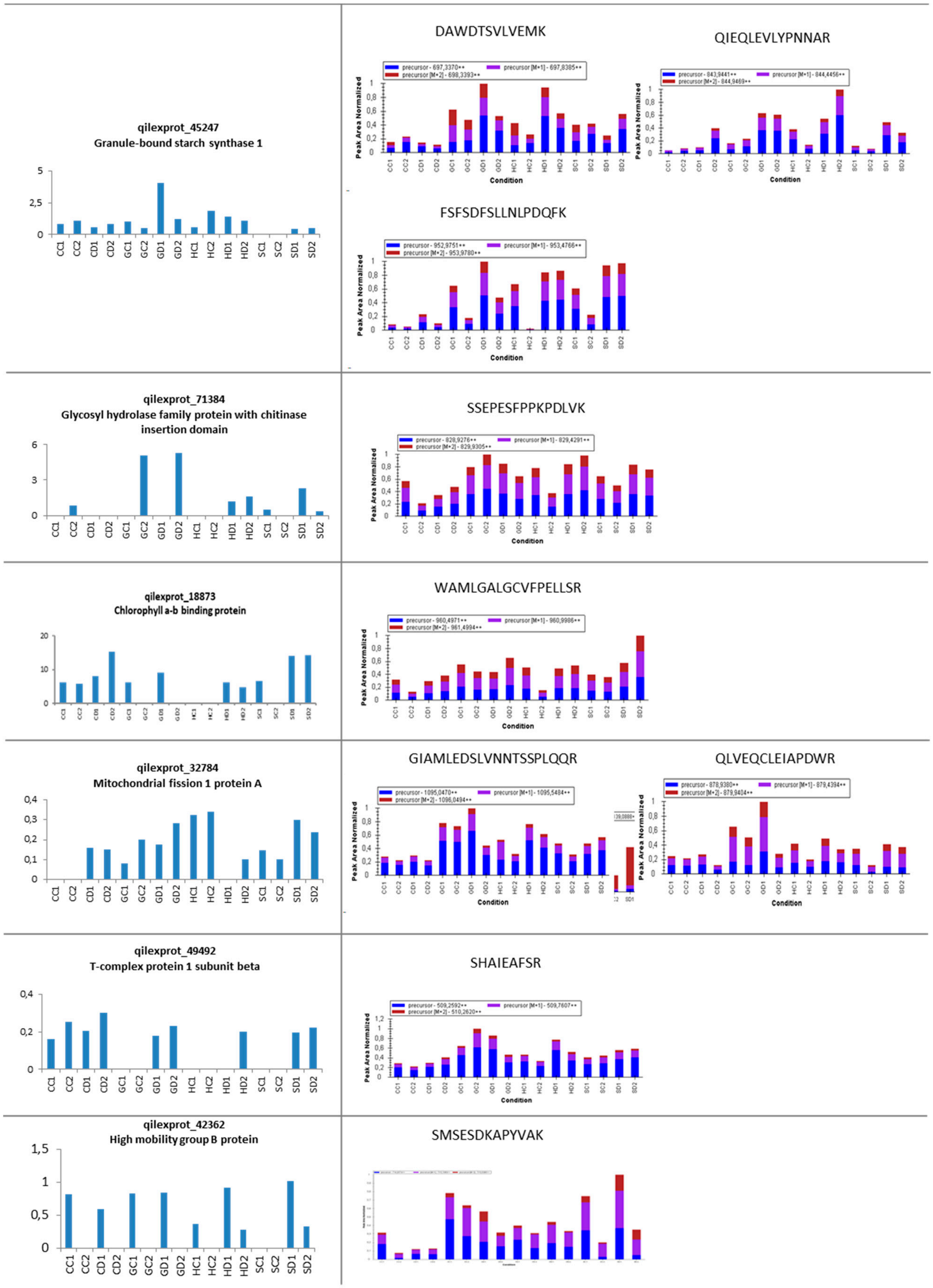

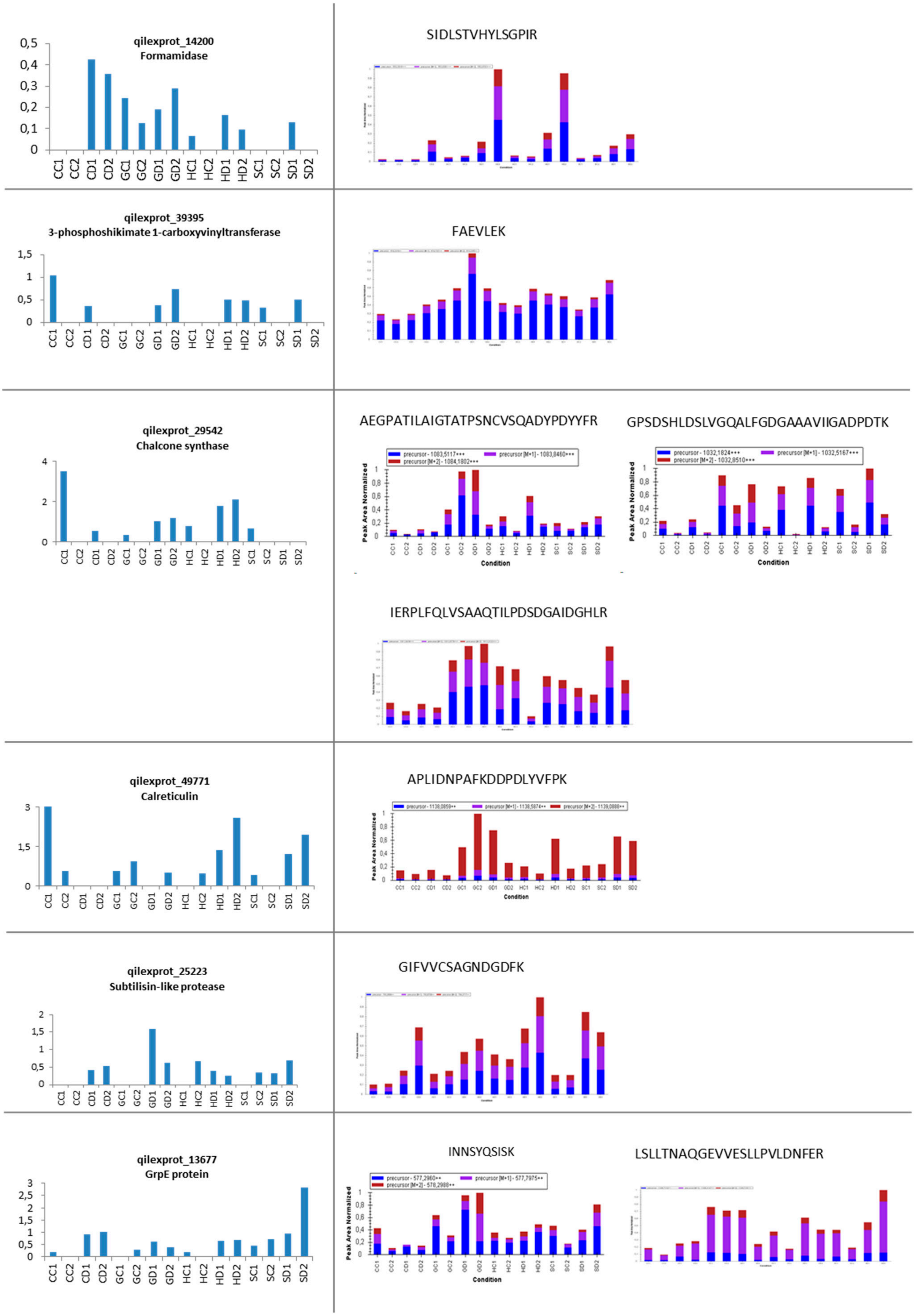

qilexprot_14200
Formamidase
SIDLSTVHYLSGPIR
qilexprot_39395
3-phosphoshikimate 1-carboxyvinyltransferase
FAEVLEK
qilexprot_29542
Chalcone synthase
AEGPATILAIGTATPSNCVSQADYPDYYFR
GPSDSHLDSLVGQALFGDGAAAVIIGADPDTK
IERPLFQLVSAAQTILPDSDGAIDGHLR
qilexprot_49771
Calreticulin
APLIDNPAFKDDPDLYVFPK
qilexprot_25223
Subtilisin-like protease
GIFVVCSAGNDGDFK
qilexprot_13677
GrpE protein
INNSYQSISK
LSLLTNAQGEVVESLLPVLDNFER
Peak Area Normalized
Condition
CC1 CC2 CD1 CD2 GC1 GC2 GD1 GD2 HC1 HC2 HD1 HD2 SC1 SC2 SD1 SD2

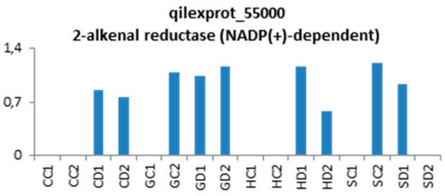

QVVLVSKE

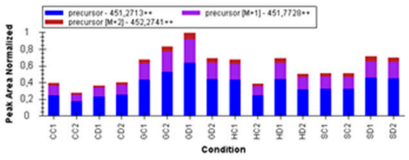

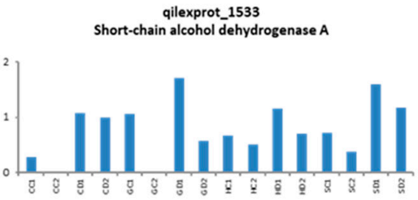

VRDVANAVLFLASDDSGFVTGLDLK

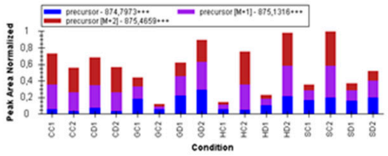

QLSTDYCMAK

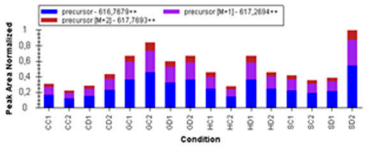

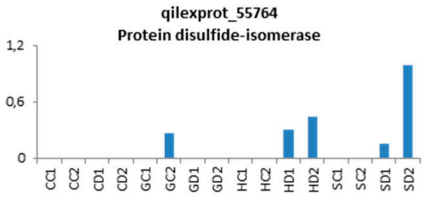

LDDDVSFYQTVNPDVAK

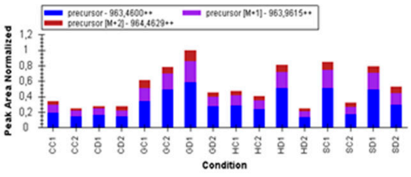

VVLGYLNSLVGPDSEELSAASK

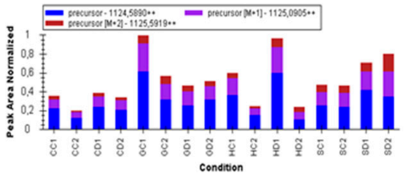

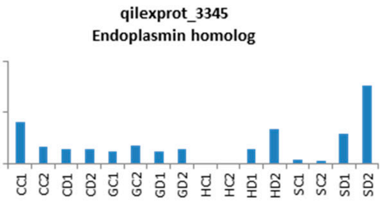

ADGAFAISEDTWNEPLGR

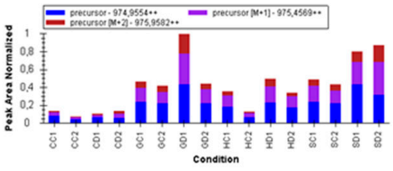

YLNFLMGLVDSDTLPLNVSR

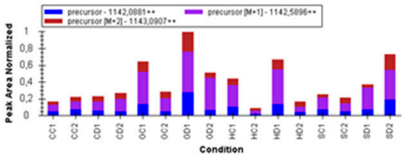

EVTEEEYTK

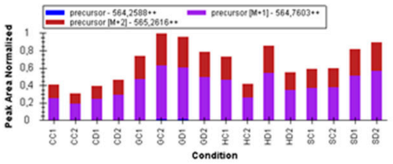

FYHSLAK

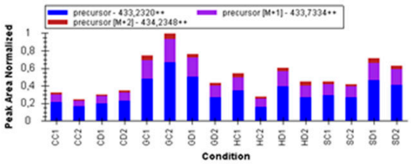

IAEEDPDEANDKDK

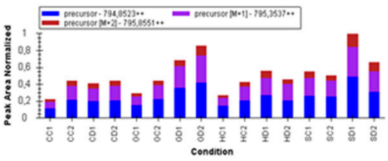

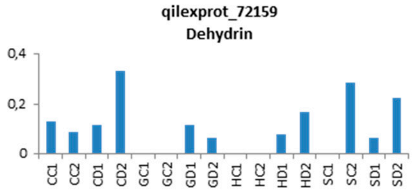

EEEMASEFEK

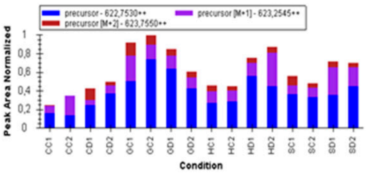

NKDEHETTTTTTPGGNEGAVESK

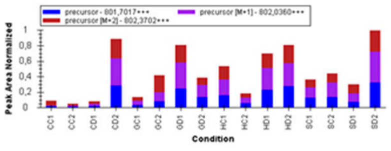

qilexprot_48029
AT3g51250/F24M12_290

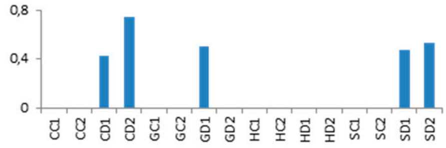

ELAENLFPEDDTVSQTPTLQSSENVLVR

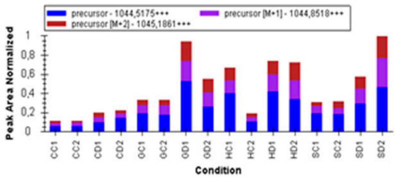

qilexprot_8871
Probable aldo-keto reductase 1

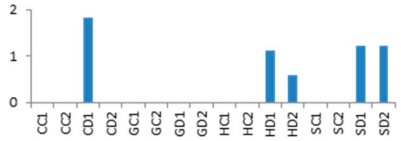

KGCTPSQLALAWVHHQGK

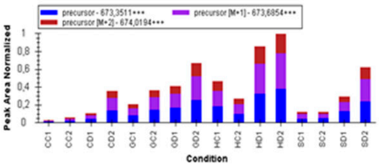

qilexprot_19464
Oligouridylate-binding protein 1B

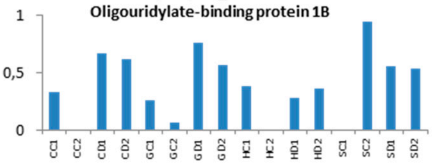

SVVELTNGSSEDGK

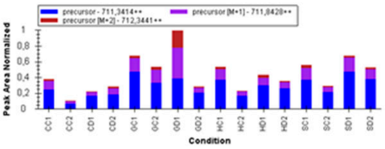

VNWAYASGQR

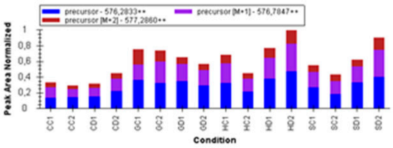

qilexprot_70616
BnaC03g49780D protein

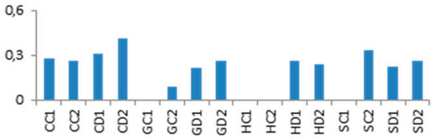

LITVTASENPDSR

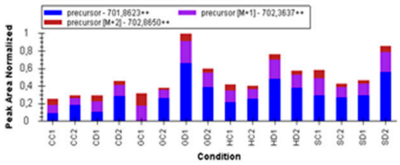

qilexprot_26698
Zinc finger protein VAR3

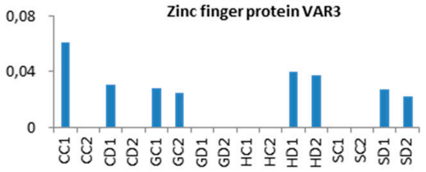

SVASNAIEWTGNASGSSVPDK

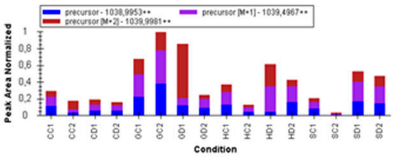

DKPESDGADLANK

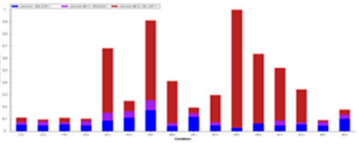

qilexprot_2527
Myb domain containing transcription regulator

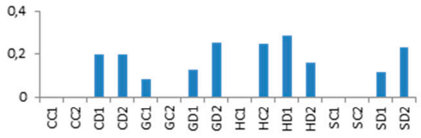

IEDIDAYAPK

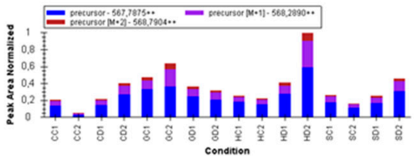

qilexprot_7552
Proliferation-associated protein 2G4

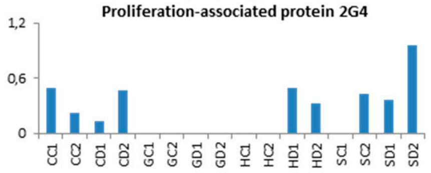

ALQLVVSECKPK

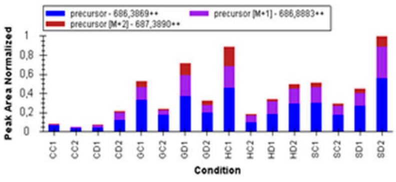

IVDVCEIGDSFIR

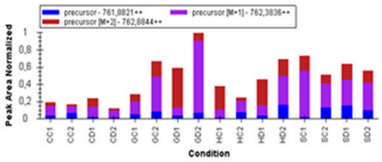

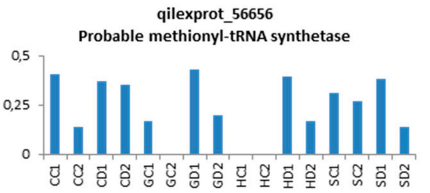
qilexprot_56656
Probable methionyl-tRNA synthetase
0,5
0,25
0
CC1 CC2 CD1 CD2 GC1 GC2 GD1 GD2 HC1 HC2 HD1 HD2 SC1 SC2 SD1 SD2

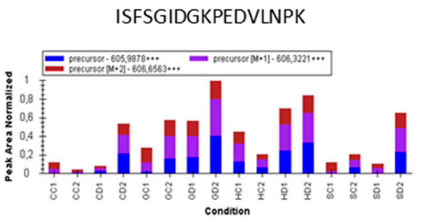
ISFSGIDGKPEDVLNPK
precursor - 605,9878+++
precursor [M+1] - 606,3221+++
precursor [M+2] - 606,6563+++
Peak Area Normalized
Condition

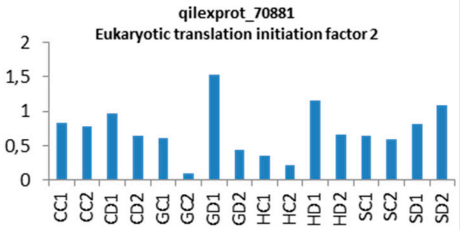
qilexprot_70881
Eukaryotic translation initiation factor 2
2
1,5
1
0,5
0
CC1 CC2 CD1 CD2 GC1 GC2 GD1 GD2 HC1 HC2 HD1 HD2 SC1 SC2 SD1 SD2

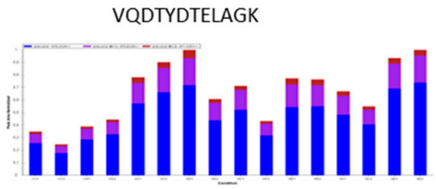
VQDTYDTELAGK

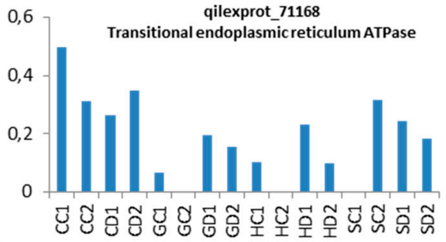
qilexprot_71168
Transitional endoplasmic reticulum ATPase
0,6
0,4
0,2
0
CC1 CC2 CD1 CD2 GC1 GC2 GD1 GD2 HC1 HC2 HD1 HD2 SC1 SC2 SD1 SD2

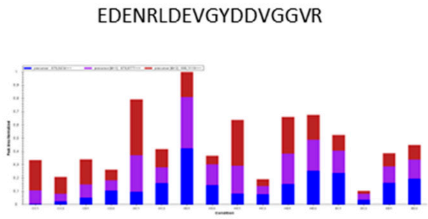
EDENRLDEVGYDDVGGVR

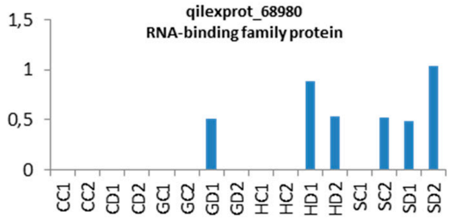
qilexprot_68980
RNA-binding family protein
1,5
1
0,5
0
CC1 CC2 CD1 CD2 GC1 GC2 GD1 GD2 HC1 HC2 HD1 HD2 SC1 SC2 SD1 SD2

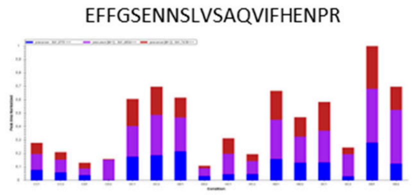
EFFGSENNSLVSAQVIFHENPR

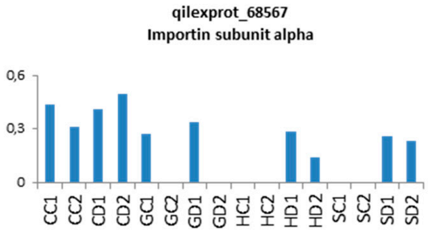
qilexprot_68567
Importin subunit alpha
0,6
0,3
0
CC1 CC2 CD1 CD2 GC1 GC2 GD1 GD2 HC1 HC2 HD1 HD2 SC1 SC2 SD1 SD2

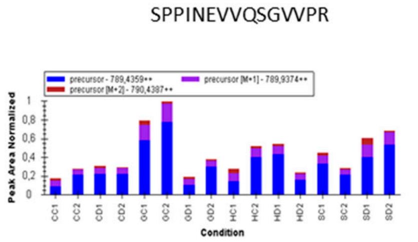
SPPINEVVQSGVVPR
precursor - 789,4359++
precursor [M+1] - 789,9374++
precursor [M+2] - 790,4387++
Peak Area Normalized
Condition

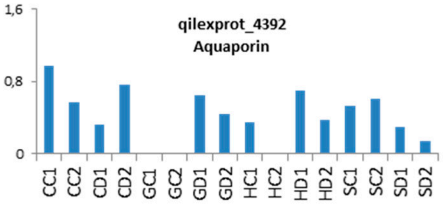
qilexprot_4392
Aquaporin
1,6
0,8
0
CC1 CC2 CD1 CD2 GC1 GC2 GD1 GD2 HC1 HC2 HD1 HD2 SC1 SC2 SD1 SD2

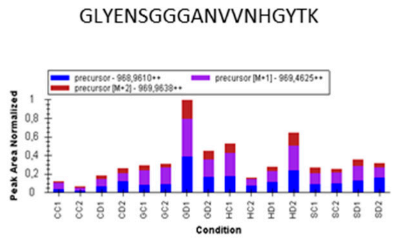
GLYENSGGGANVVNHGYTK
precursor - 968,9610++
precursor [M+1] - 969,4625++
precursor [M+2] - 969,9638++
Peak Area Normalized
Condition

Supplementary Figure S5

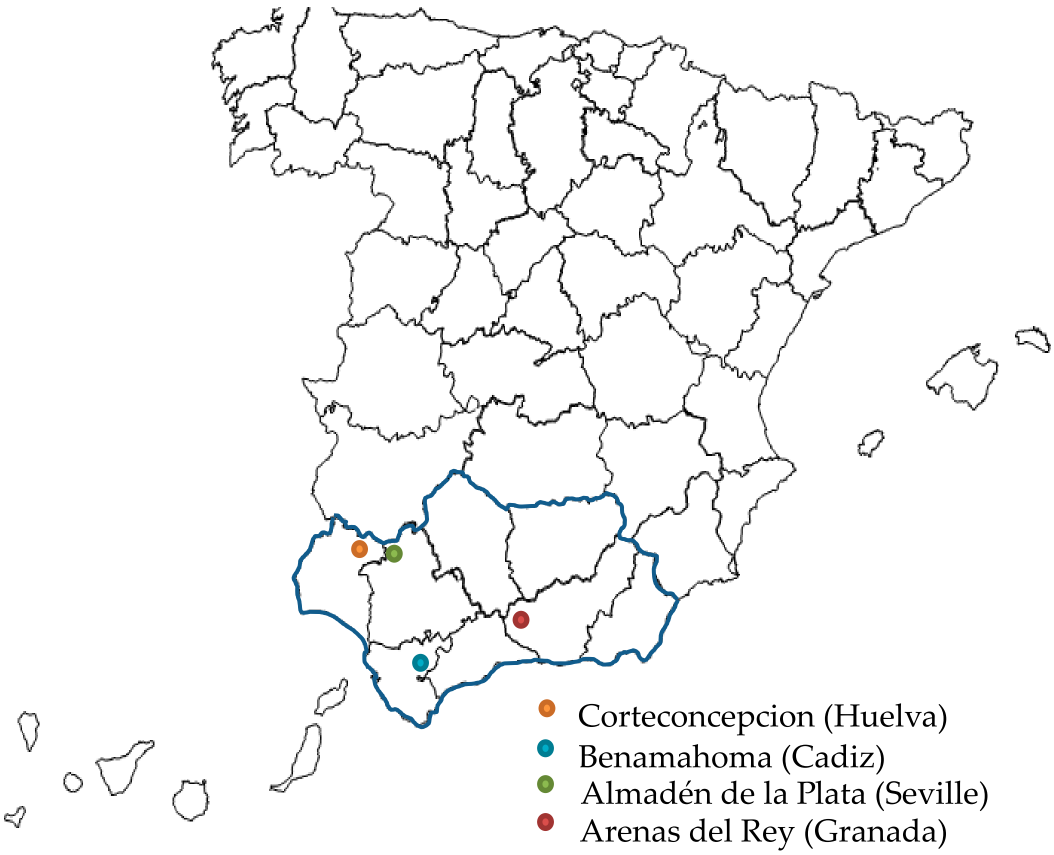

Supplementary Figure S6

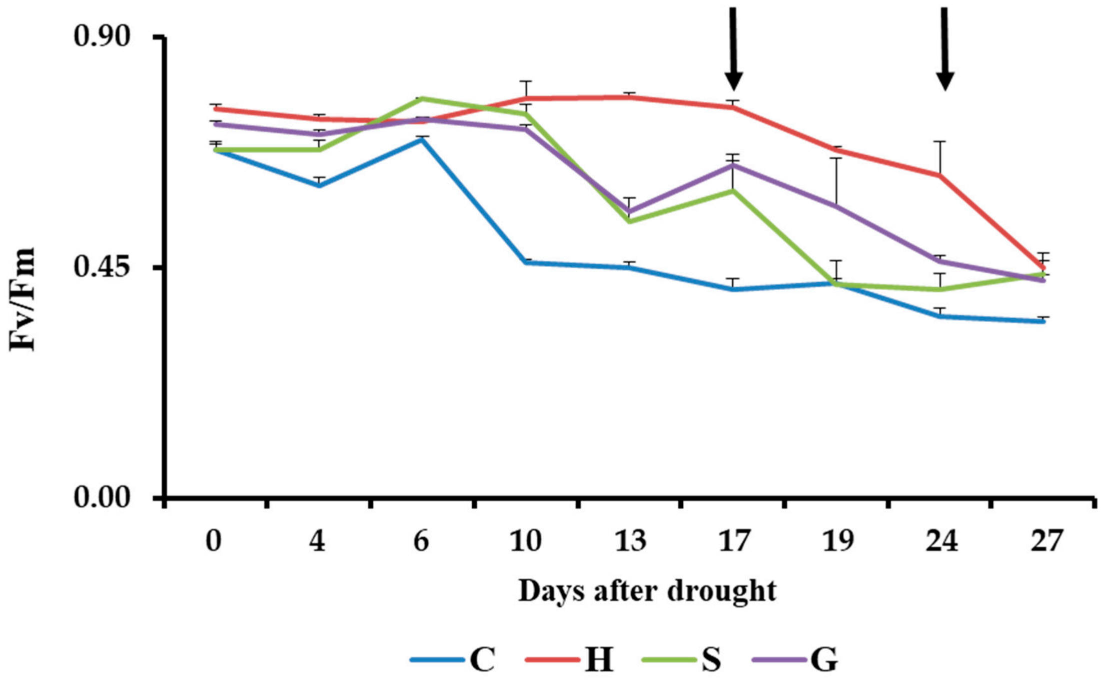

Supplementary Figure S7

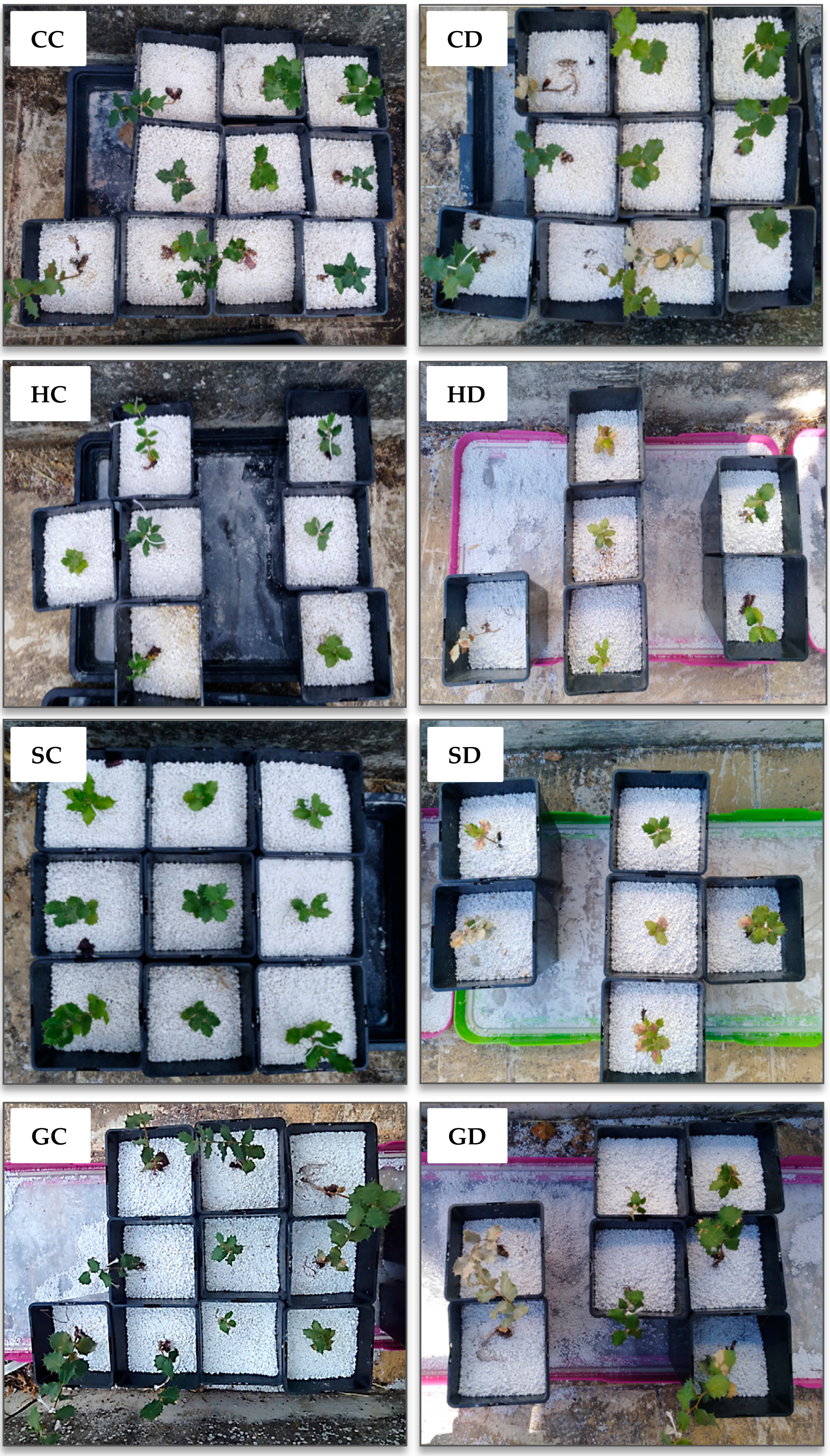

Supplement: Supplementary file 1 [file ijms-22-03191-s001.pdf]
